# Rotational Effects of Nanoparticles for Cooling down Ultracold Neutrons

**DOI:** 10.1038/srep44070

**Published:** 2017-03-15

**Authors:** Xiaoqing Tu, Guangai Sun, Jian Gong, Lijuan Liu, Yong Ren, Penglin Gao, Wenzhao Wang, H. Yan

**Affiliations:** 1 Institute of Nuclear Physics and Chemistry, China Academy of Engineering Physics, Mianyang, 621999, People’s Republic of China; 2 State Key Laboratory Cultivation Base for Nonmetal Composites and Functional Materials, Southwest University of Science and Technology, Mianyang, 621010, People’s Republic of China

## Abstract

Due to quantum coherence, nanoparticles have very large cross sections when scattering with very cold or Ultracold Neutrons (UCN). By calculating the scattering cross section quantum mechanically at first, then treating the nanoparticles as classical objects when including the rotational effects, we can derive the associated energy transfer. We find that rotational effects could play an important role in slowing down UCN. In consequence, the slowing down efficiency can be improved by as much as ~40%. Since thermalization of neutrons with the moderator requires typically hundreds of collisions between them, a ~40% increase of the efficiency per collision could have a significant effect. Other possible applications, such as neutrons scattering with nano shells and magnetic particles,and reducing the systematics induced by the geometric phase effect using nanoparticles in the neutron Electric Dipole Moment (nEDM), are also discussed in this paper.

Slowing down neutrons is a crucial problem in both fundamental physics and condensed matter physics. For fundamental neutron physics, the slower the neutrons, the longer the measurement time. Therefore more precise measurements can be made for the slower neutrons. Neutrons with energy lower than 10^−7^ eV are classified as Ultracold Neutrons(UCN) which can be confined by gravity and form quantum bound states^1^. UCN experiments can help us know more about the fundamental physical laws in many ways. For example, by precisely measuring the gravitational quantum states of neutrons, constraints can be imposed on the coupling between the hypothetical chameleon fields and ordinary matters^2^,^3^. Moreover, the study of neutron lifetime can further our understanding of weak interaction between hadrons^4^,^5^,^6^,^7^, the measurement of nEDM can help in solving the CP violation problem^8^, the neutron *β*-decay experiment can be implemented to measure the Axial-Vector weak coupling constant^9^, and the spin precession of UCN has already been used to test the Lorentz invariance^10^. In condensed matter physics and other interdisciplinary field, UCN have been used in studying the slow kinetic process of macromolecule, fundamental biological science, biomedicine and biopharming. The length scale that the neutron can probe depends on its wavelength. The larger the wavelength, the larger the neutron probing scale. Small Angle Neutron Scatter(SANS), Ultra Small Angle Neutron Scattering(USANS) and Spin Echo Small Angle Neutron Scattering(SESAME) have been developed to probe large scale structures of various samples by measuring small angle scattering precisely. How to obtain more UCN is important in fundamental science, practical engineering, and industrial applications^11^,^12^,^13^,^14^,^15^,^16^, etc. Slowing down neutrons effectively is the key problem since the day when neutron was discovered.

For typical thermal neutron sources, the percentage of UCN is extremely small. At neutron temperature of 300 K, the population ratio of UCN is ~10^−11^, and it can only be increased to ~10^−9^ when the neutron temperature is cooled down to 20 K. Obviously, obtaining UCN by directly selecting it from a wide energy spectrum is not efficient. There are other methods such as using gravity or reflection from a moving surface to decelerate a beam of neutrons^17^. However all these methods can not increase the original phase density in the primary moderator^18^.

Nesvizhevsky *et al*. have proposed a novel moderator scheme for UCN using nanoparticles^19^, in which nano spheres made by low neutron-absorption materials are immersed in the liquid helium at temperature close to 0 K. The nanoparticles are in thermal equilibrium with the superfluid helium. Hence there is no friction and the nanoparticles can move freely and scatter with UCN intensively in the moderator, which we will illustrate later by a detailed example. Nesvizhevsky *et al*. first calculated the neutron scattering cross section quantum mechanically. During that process, the neutron energy can be transferred to the translational movement of the nanoparticles. The energy loss of the neutron to the nanoparticle was obtained by using the principle of energy-momentum conservation. It was found that under some specific experimental conditions, this method might increase the ultracold neutron density by several orders of magnitude^19^,^20^. In comparison with other methods in which UCN are obtained by selecting neutrons within a very narrow fraction of the whole energy spectrum, this new method can increase the phase space density of UCN.

However, rotational effects are neglected in the previous study^19^. Intuitively, rotational degrees of freedom of nanoparticles could play a non-negligible role. In this paper, we try to further this interesting idea by including rotational effects, and the same conditions assumed in ref. 19 are followed, i.e., free nanoparticles are in thermal equilibrium with superfluid helium at 0 K, no internal degrees of freedom of nanoparticles are excited for the neutron energy under consideration, etc. Thus the limitations of applicability in the previous study in ref. 19 are also the same in our work. The remainder of this article is organized as follows. We first illustrate quantum coherence effect for neutron scattering by a simple example. Then we present our methods and results in which the rotational effects of nanoparticles are included. Other possible applications are also discussed, such as neutron scattering with nano shells and magnetized nanoparticles, and reducing the geometric phase effect which is one of the most important systematics of the nEDM experiment. Finally, we present the conclusion of this work.

## Quantum Coherence

Due to the similarity between the neutron wavelength and the nanoparticle size, cold neutrons and UCN interact strongly with nanoparticles because of quantum coherence. Simple cases are illustrated here. First, we suppose the slow neutrons described by a plane wave are scattered by a single nucleus fixed at the origin. Then the scattered neutron wave can be expressed as follows during this low energy process^18^,^21^,^22^,^23^,^24^:


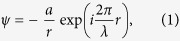


where *a* is the scattering length of neutrons in the nucleus, *λ* denotes the de Broglie wavelength of the incident neutron, and *r* is the distance from the origin. It is easy to obtain the total scattering cross section:


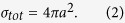


Then we come to another situation, in which two nucleus of the same type fixed at different positions 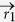 and 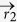 are scattered by neutrons. The scattered wave is:


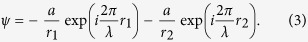


Since the wavelength of UCN is much larger than the distance between the nucleus, i.e., 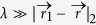, the scattered wave can be approximated as:


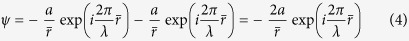


where 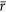 is the average of *r*_1_ and *r*_2_. The total scattering cross section in this case is given by:


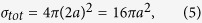


which is four times of that associated with single nucleus. In short, due to the very large wavelength of UCN compared with nano-sized particles, quantum coherence plays a crucial role. It produces a quadric increment for the total scattering cross section as the nucleus number of the nanoparticle increases. For instance, if a *C*_60_ atom is scattered by UCN, its total scattering cross section is 3600 rather than 60 times of that of a single carbon atom.

Based on these facts, UCN will be scattered strongly by the nano-sized particle immersed in the low temperature superfluid helium moderator. Therefore neutrons can be moderated by these nanoparticles.

## Mean Energy Loss per Collision between a Neutron and a nanoparticle

In ref. 19, the total cross section of a neutron scattered by a fixed nanoparticle was calculated quantum mechanically using the first order Born approximation. During the collision, the neutron energy is transferred to the translational movement of the nanoparticles. The energy loss of the neutron to the nanoparticles is obtained classically by simply applying the energy-momentum conservation. The simplicity and elegance of the method are inspiring. We try to include the rotational effects of the nanoparticles in a similar manner, i.e., first calculating the scattering cross section quantum mechanically, then studying the rotational effects of the nanoparticles classically.

**Figure 1:**
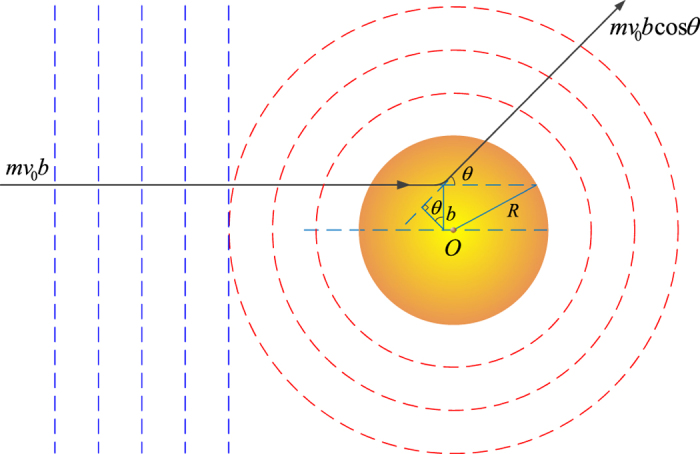
neutron wave scattered by a nanoparticle.

We first calculate the cross section of neutrons scattering with nanoparticles by using the first order Born approximation. Note although a more precise calculation of the total scattering cross section by using the partial wave method has been done in ref. 25, only a ~10% difference from the Born approximation is observed. Next, to obtain the energy carried away by the rotational degrees of nanoparticles, we treat the latter as classic objects. As illustrated in Fig. 1, an incoming neutron with impact parameter *b* is scattered by a nano sphere of radius *R*. Classically, only if the impact parameter is within the range of the particle size, can a collision occur. The energy, momentum and angular momentum will be redistributed between the two objects. Since the translational energy transfer was already solved, here we only consider the results due to the rotational degrees of freedom.

According to the law of angular momentum conservation,


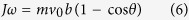


where *J* is the momentum of inertia, *m* the neutron mass, *v*_0_ the incident neutron velocity, *ω* the angular velocity of the nano sphere, and *θ* the scattering angle. Applying the law of energy conservation, the rotational energy carried by the sphere is expressed as:


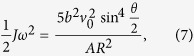


where *A* is the nano sphere mass in units of neutron mass. The ratio of rotational energy transfer is easily obtained:


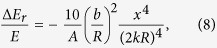


where 
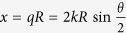
, 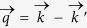 is the scattering wave vector, 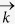 is the incident wave vector, and 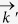 is the final wave vector. The collision is elastic in the center-of-mass frame, so we have 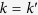.

**Figure 2:**
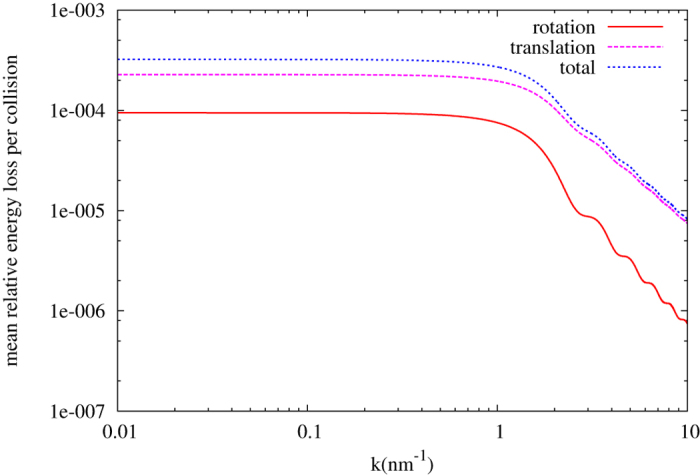
Translation, rotation, and total relative mean energy loss per collision, as a function of neutron wave vector, for the diamond nanoparticle with radius of 1 nm.

Substituting the scattering amplitude into Eqn. (8), the mean energy loss due to rotational degrees can be derived as:


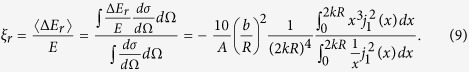


where *j*_1_(*x*) is the first order spherical Bessel function. We calculate the above integrals numerically by using the Simpson’s method^26^. When calculating the rotational-degree-caused energy transfer, the problem is considered classically, in which the neutron is considered as a point-like particle. Only if the impact parameter is within the radius of the nanoparticle, will a collision happen. In this classical situation, the average impact parameter is obviously 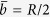 for incident neutrons when collisions occur. The total energy loss is the sum of the energy loss due to the translation and rotation movements. We present the behavior of the energy loss ratio due to rotational effects in Fig. 2 for a diamond nanoparticle with radius of 1 nm.

When the incident neutron wave vector is much smaller than the nanoparticle size, i.e., 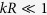:


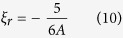


while in this case the translational energy transfer rate is:


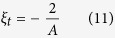


**Figure 3:**
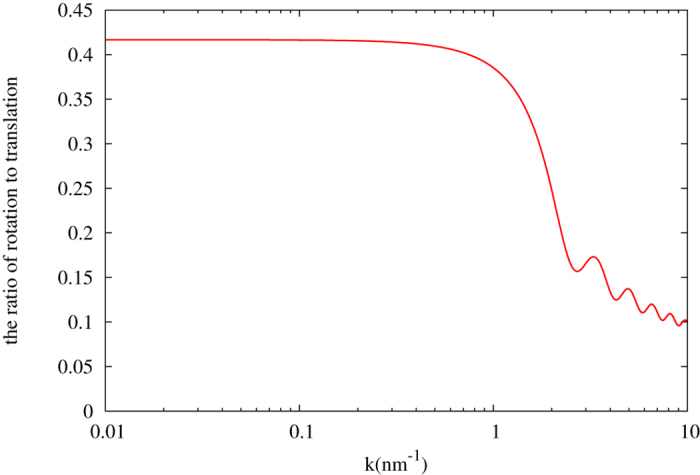
The ratio of energy loss due to rotation degrees of freedom to translation of the 1 nm dimond nano sphere.

It is easy to see that under long wavelength limit, rotational degrees of freedom of the nanoparticles carry as much as 5/12, i.e. ~40% amount of energy as the translational degrees of freedom. As we can see from Fig. 3, when the rotational effects are taken into account, the energy transfer efficiency is increased by as much as 40%. To slow down neutrons as much as possible, it is important to increase the energy transfer efficiency for each collision between neutrons and the moderator. Typically, hundreds or even thousands of collisions are required for neutrons to be in thermal equilibrium with the moderator, so a 40% increase of the efficiency per collision can be significant.

## Neutrons Scattered by a Nano Shell

The average relative energy loss is inversely proportional to the mass of nanoparticles. Higher moderating efficiency is expected when the mass of the scatterer becomes smaller. Therefore, we turn to consider the nano shell, of which the mass is much smaller than that of the nanoparticle in the same volume. Here we calculate the associated moderating efficiency. For simplicity, we only consider single layer shells. The phenomenological potential for the interaction of a neutron with a nano shell can be express as:


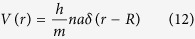


where *h* is the Planck’s constant, *n* the neutron surface density of the shell and *R* the shell radius.

Using the first order Born approximation, the total scattering cross section is found to be:


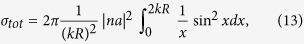


The total mean relative energy loss per collision is derived as


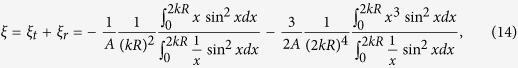


where *A* is the mass of nano shell, again in units of the neutron mass. As a shell has a relatively larger initial momentum than a sphere since the former concentrates its mass on surface, we expect that rotational effects are less efficient. In fact, if we take long wavelength limit, the ratio is


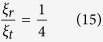


which is ~17% less than the sphere case.

**Figure 4:**
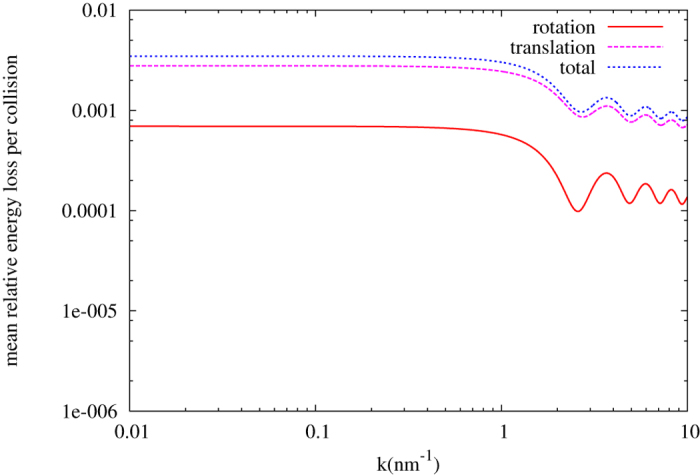
Translation, rotation, and total relative mean energy loss per collision, as a function of neutron velocity, for C_60_.

For practical purposes, we consider using *C*_60_, of which the radius is 0.71 nm, to moderate UCN. By applying Eqn. (13), we found that the total scattering cross section of *C*_60_ is 3600 times of a single carbon nucleus. The derived energy loss per collision of *C*_60_ is shown in Fig. 4.

Though mean energy loss per collision is proportional to 1/*A*, i.e., inversely proportional to the total nucleus number in the nanoparticle, the neutron scattering cross section is in quadratic relation to the nucleus number. If we only take the scattering effects into consideration, higher neutron cooling efficiency is expected for larger nanoparticles. However, the neutron absorption cross section increases linearly with the nucleus number. It will be an optimization problem to balance all these factors.

## Neutrons Scattered by a Magnetized nanoparticle

It is known that neutrons can interact with magnetic particles since they have a magnetic moment. Hence it is natural to consider the magnetic effects for some specific nanoparticles. According to ref. 21, magnetic scattering has no interference with the nuclear scattering. The magnetic scattering cross section includes spin states transitions and for the elastic case it can be expressed as:


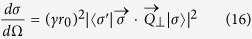


where *r*_0_ is the classical radius of electrons, and *γ* is the gyromagnetic ratio of neutrons, 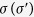 refers to the spin state, 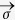 are the Pauli matrices and:


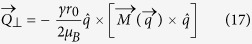


where 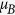 the magnetic moment of electrons, 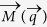 is the Fourier transformation of the nano sphere magnetization 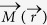.

**Figure 5:**
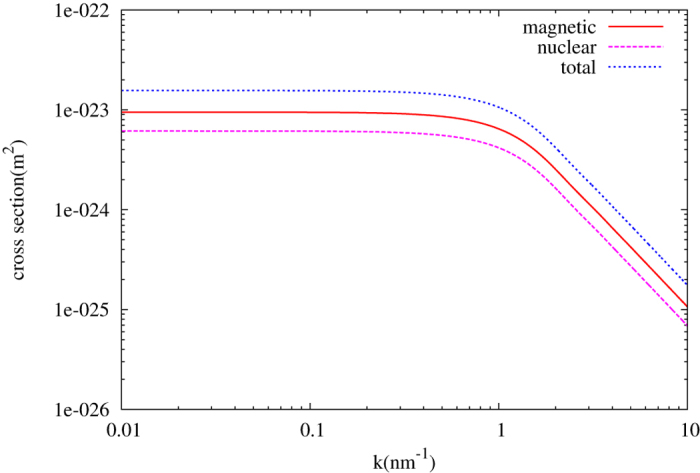
Nuclear, magnetic and the total scattering cross sections of Co nanoparticle with radius of 1 nm.

Using Eqn. (16), the total cross section of a neutron scattered by a magnetized nanoparticle is found to be:


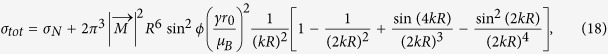


where σ_*N*_ is the nuclear scattering cross section of the nanoparticle, *ϕ* the angle between the scattering wave vector and the magnetization. For atoms with strong magnetism, we expect that the magnetic scattering might be important. For example, the magnetic scattering length of the Co atom is even larger than its nuclear scattering length, then its magnetism may also serve to slow down neutrons. The magnetic, nuclear and total cross sections are shown in Fig. 5.

## Possible Application in the nEDM Experiments

There are other possible applications of nanoparticles in fundamental neutron physics. In the nEDM experiment, the geometric phase effect is considered to be a major systematic error^27^, which is due to the correlation between the velocity and the position time series of the UCN^28^. When using ^3^He atoms as the co-magnetometer, it was found that this geometric phase effect get suppressed due to randomizations caused by collisions between ^3^He atoms and liquid ^4^He. It was also noticed that the diffusive reflections of UCN by the wall can reduce this effect^28^. However, this effect can not bring great benefits here since the neutron has a negligible cross section when scattering with ^4^He. If we introduce nanoparticles into the liquid ^4^He, not only the neutrons can be further slowed down, but also the geometric phase effect caused by scattering neutrons is reduced.

## Conclusion and Discussion

Due to quantum coherence, nano-sized particles have very large cross sections when scattered with very cold and ultracold neutrons. Neutrons with large wavelength will strongly interact with the nanoparticles immersed in the superfluid helium, which consequently take away energy of the incident neutrons. Based on this fact, neutrons could be slowed down if the temperature of nanoparticles is lower than that of neutrons. Previously, only translational energy is considered within this new moderating mechanism. By taking into account of the rotational effects, we find that neutron-moderating efficiency might be increased by as much as 40% accordingly. One of the most important advantages of this moderator scheme is its simplicity. For the superthermal liquid helium UCN moderator used by several nEDM experiments^29^,^30^,^31^, if their operating temperature can be lowered down to ~mK, the moderator scheme discussed in this paper could be realized simply by adding some nanoparticles. Since many tests and experiments have been done for the superthermal UCN sources operating at temperatures of a few hundreds mK, we would expect the main technology difficulty might be cooling the liquid helium inside the moderator down to ~mK. The temperature required is just the practical lower limit of the commercially available dilution refrigerators. Practically, it would be difficult to realize the new moderator scheme but it is not impossible. We further derive the scattering cross section and moderating efficiency of neutrons scattered by a nano shell made by *C*_60_ as an example. We also consider using the magnetic nanoparticles to slow down neutrons, and give the associated scattering cross section. We find that nanoparticles could be applied to the nEDM experiment not only to slow down neutrons but also reduce a systematic error.

## Additional Information

**How to cite this article:** Tu, X. *et al*. Rotational Effects of Nanoparticles for Cooling down Ultracold Neutrons. *Sci. Rep.* **7**, 44070; doi: 10.1038/srep44070 (2017).

**Publisher's note:** Springer Nature remains neutral with regard to jurisdictional claims in published maps and institutional affiliations.
